# Integration of feature vectors from raw laboratory, medication and procedure names improves the precision and recall of models to predict postoperative mortality and acute kidney injury

**DOI:** 10.1038/s41598-022-13879-7

**Published:** 2022-06-17

**Authors:** Ira S. Hofer, Marina Kupina, Lori Laddaran, Eran Halperin

**Affiliations:** 1 Department of Anesthesiology and Perioperative Medicine, David Geffen School of Medicine at UCLA, 757 Westwood Plaza, Los Angeles, CA 90095 USA; 2 Department of Anesthesiology, Pain and Perioperative Medicine, Icahn School of Medicine at Mount Sinai, New York, USA; 3 Frank H. Netter MD School of Medicine of Quinnipiac University, North Haven, USA; 4 Department of Computer Science, University of California, Los Angeles, CA USA; 5 Department of Anesthesiology and Perioperative Medicine, University of California, Los Angeles, CA USA; 6 Department of Human Genetics and Biomathematics, University of California, Los Angeles, CA USA

**Keywords:** Preclinical research, Machine learning

## Abstract

Manuscripts that have successfully used machine learning (ML) to predict a variety of perioperative outcomes often use only a limited number of features selected by a clinician. We hypothesized that techniques leveraging a broad set of features for patient laboratory results, medications, and the surgical procedure name would improve performance as compared to a more limited set of features chosen by clinicians. Feature vectors for laboratory results included 702 features total derived from 39 laboratory tests, medications consisted of a binary flag for 126 commonly used medications, procedure name used the Word2Vec package for create a vector of length 100. Nine models were trained: baseline features, one for each of the three types of data Baseline + Each data type, (all features, and then all features with feature reduction algorithm. Across both outcomes the models that contained all features (model 8) (Mortality ROC-AUC 94.32 ± 1.01, PR-AUC 36.80 ± 5.10 AKI ROC-AUC 92.45 ± 0.64, PR-AUC 76.22 ± 1.95) was superior to models with only subsets of features. Featurization techniques leveraging a broad away of clinical data can improve performance of perioperative prediction models.

## Introduction

The last several years have seen an explosion in the number of papers using machine learning (ML) techniques to predict a variety of perioperative outcomes. Models have been successfully developed to predict key outcomes such as hypotension^1,2^, mortality^3–6^, readmission^7^, and acute kidney injury (AKI)^4,8–11^. As a group, these papers have proven the underlying hypothesis that ML techniques can be applied to healthcare data to predict outcomes.

Fortunately, negative outcomes in the perioperative setting are relatively rare. For example, the overall incidence of postoperative mortality is roughly 1–4%^12^, and the rate of postoperative AKI is roughly 12%^13^. As a result, most models that have been published report relatively high accuracy and areas under the receiver operating characteristic curve (ROC AUC), while having an area under the precision recall curve (PR AUC) that are lower^3–5,14^. This is more than a theoretical limitation. The successful implementation of ML models into clinical practice requires the successful identification of the rare event to the exclusion of other cases. To use a concrete example if a model is to be used to change the decision of surgery, the positive predictive value (precision) must be very high, otherwise large numbers of patients will receive the incorrect treatment.

One limitation of many of the ML models that have been published, especially in the perioperative space, is that they rely on a limited number of features, hand-selected by a clinician to predict the outcome of interest. While this has proven effective, it also serves to leave a large amount of information from the electronic health record (EHR) out of the model, thus potentially limiting the model’s predictive power.

In this manuscript we hypothesized that creating feature vectors from a wide variety of data types in the EHR would improve model performance. In particular, we hypothesized that creating a feature vector for each of the last six months of laboratory values, patient medications and the type of procedure (as specified in the procedure name) would improve the performance of models trained to predict postoperative mortality and AKI as compared to a baseline feature set. As a primary outcome we look at the area under the Receiver Operating Characteristic (ROC) and Precision-Recall (PR) curves and as a secondary outcome we look at accuracy, precision and recall and various thresholds.

## Methods

Data were extracted from the Perioperative Data Warehouse (PDW), a custom-built data warehouse containing all patients who have undergone surgery at the University of California Los Angeles (UCLA) Health since the implementation of our EHR (EPIC Systems, Madison, WI) in March 2013. The PDW has been previously described^15,16^. Briefly, in the first stage, data are extracted from EPIC’s Clarity database into 29 tables organized to facilitate usage. In the second stage, these data are used to populate a series of measures and metrics such as procedure duration, readmissions, admission International Classification of Diseases (ICD) codes, and postoperative outcomes^17–19^. All data used for this study were obtained from this data warehouse, and institutional review board approval (18-002053) was obtained from the UCLA Office of the Human Research Protection Program, including exemption from written informed consent, for this retrospective review. All methods were performed in accordance with relevant guidelines and regulations.

### Determination of outcome variables—mortality and AKI—from EHR data

Postoperative mortality was defined as death during the same hospitalization as the surgery as identified by either (1) a death date is noted in the EHR between hospital admission and discharge or (2) a postoperative discharge status of expired, presence of a “death note” by a treating provider, and the lack of future admissions or encounters for the patient in the EHR. Postoperative AKI was defined based upon the Acute Kidney Injury Network (AKIN) criteria creatinine criteria^20^. The baseline creatinine was taken to be the most recent serum creatinine prior to surgery. The postoperative creatinine was the highest creatinine value within 48 h of surgery. If either the preoperative or postoperative creatinine was missing, the value was set to NULL. A value of 0 was used to denote no AKI while any AKIN stage 1 or above was denoted as a 1 (i.e. this was set to a binary variable). It would have been possible to create a multi-class prediction algorithm to predict the actual AKI class, as opposed to the binary classification, however we chose to use the more simplistic binary classification in order to keep the focus of the analysis on the featurization techniques.

### Inclusion and exclusion criteria

The inclusion data set was surgeries with general anesthesia that occurred between April 1, 2013 and July 2021 that were performed at the Ronald Reagan UCLA Medical Center and Santa Monica Medical Center hospitals. Patients were filtered by their class, selecting only inpatient, same day hospitalization, emergency care and overnight recovery patients (i.e. those that spent at least one night in the hospital). Patients aged less than 18 years old or older than 89 years old were excluded from the dataset due to the institutional restrictions on data security. Cases were excluded if they had an ASA physical status score of 6 (indicating organ donors).

**Figure 1:**
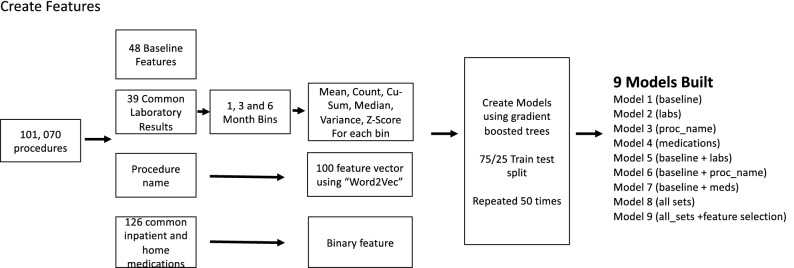
Study design.

Figure 1 demonstrates the overall study design.

### Model input features

In this manuscript, four sets of input features were defined depending on their characteristics: (1) baseline features including basic patient information and surgery specifications, (2) the most recent laboratory tests obtained before the surgery, (3) procedure description, and (4) medications taken.

### Baseline feature vector

The features used in the baseline model were based on previous work by our group predicting postoperative mortality both before and after surgery^3–5^. For the purposes of this analysis, we removed those features that would be redundant with the additional feature vectors (see below). For example, lab results were removed from the feature sets used in those models because we created a separate, more comprehensive feature vector, comprised only of the labs. The list of features included in this group is in Supplementary table 1A.

### Laboratory result feature vector

A set of 39 commonly used laboratory results were extracted from the EHR (see Supplementary Table 2A for a complete list). These results were chosen because they are common preoperative tests (i.e. included in complete blood count, comprehensive metabolic panels or coagulation panels). Test all results were then binned in 6 months before surgery, 3 before months, and 1 month prior to surgery. Then, for each laboratory test bin the following descriptive statistics were calculated: total number of tests, cu-sum, median, variance, mean, standard score (z-score). The goal of the cu-sum was to incorporate a measure of temporal change into the descriptive statistics. The standard score (or z-score) is the number of standard deviations at which the mean of a patient's test results for each laboratory is higher or lower than the mean of that test for all patients. Thus, for each surgery we had a vector of 702 features (39 laboratory results, 3 bins, 6 descriptive statistics per bin).

### Procedure name embedding

As part of our research, we experimented with the inclusion of clinical text data in the form of procedure names as the model inputs. Administrative codes, such as CPT codes are only available after surgery (patient discharge), thus we focused on representing the procedure name using a numerical vector available before surgery. The procedure name, as booked by the surgeon, consists of a string with a variable number of words. The number of unique words contained in all procedures names was 22,003 in the training dataset. In order to include the procedure name in the prediction model, we applied word embedding; a common method for representing words, typically in the form of a real-valued vector that encodes the meaning of the word such that the words that are closer in the vector space are expected to be similar in meaning^21^. We trained word embeddings on the clinical text data to allow the model to understand a clinical context using class Word2Vec from Gensim library^22^. In order to train the model, each procedure name is broken into words (tokens). The Word2Vec model takes a list of tokens for each sentence as input and returns a set of numeric vectors as output using a two-layer neural network. The Word2Vec guide suggests the size of dimensions ranging from 100 to 1000. In this paper, the size of the numeric vector is given as a model parameter and is chosen during the hyper-parameter tuning process. The dimension of the numerical vector depends on the corpus that was trained on the names of clinical procedures. Since the size of the trained corpus is not large enough, higher values do not affect the result and increase the overall computation time. While the lowered dimensions reduced the quality of the model. In this paper a vector size of 100 was found to be optimal. The trained model was applied for each procedure name, returning a set of numeric vectors for each word. Since the procedure name contains multiple words, the length of the procedure name is variable. Thus, in order to get a single vector representation of the procedure name, we calculated the average vector of its words, which is the input to the main predictive model.

### Medications

The last type of feature we analyzed was the medications taken by a patient before surgery. A set of high use and likely clinically predictive medications was made based on clinical judgement. Medications that were taken 24 h before surgery were excluded. Medications were broken up into two different categories: given as an inpatient and taken at home. Combined medications (e.g. HYDROCODONE-ACETAMINOPHEN 5-325 MG PO TABS) were separated into single medications, and each prescription dose was calculated with normalized units (e.g. Hydrocodone 5 mg, Acetaminophen 325 mg). For each patient we created a binary vector, where each element of the vector indicated if the specific medication was taken by a patient. Then for each medication the Fisher’s Exact Test was used to determine a significant association between medication and target variable. Medications with a p-value of less than 0.05 were included in the model. In order to avoid any bias, the Fisher’s Exact Test was applied only to data in the training data set. The final vector contained a set of 126 medications (see Supplementary Table 3A for a complete list).

### Data preprocessing

Data were split training and testing datasets with the split ratio 75:25. To avoid information leakage, all patients that appeared in the test set were removed from the training set.

Categorical variables such as ethnicity, gender, etc. in clinical data were converted to a numerical representation by applying the One-Hot-Encoding algorithm that decoded each category in a binary vector. To optimize the memory usage, a memory reduction function was implemented that validated the feasibility of the data type modification. On the training dataset, this technique decreased the dataset used memory by almost 68% from 740 to 237 MB, which significantly accelerated the model training process. Because single training-test splits are subject to bias, the train test splits were done 50 times and the results were averaged.

### Model creation, training, and testing

Nine models were trained. Model 1 is the baseline model that includes only the basic features in Supplementary Table 1A. Models 2, 3 and 4 were trained only on laboratory results, procedure name embeddings, and medication features respectively. The main goal of training these models was to measure the individual contributions to the prediction model. Models 5, 6, and 7 were extensions of Models 2, 3, and 4 with added features from the baseline model to the features of the respective models (i.e. baseline + laboratory, baseline + medications, etc.). Finally, Model 8 was obtained by combining features from the previous models—1088 features in total (https://github.com/scikit-learn-contrib/boruta_py).

To simplify Model 8, we built Model 9which was designed to include the most powerful features from Model 8. For feature selection, we chose a wrapper-based feature selector Boruta ^23^. Unlike other popular feature selection algorithms, Boruta analyzes feature interactions and ranks each feature to get all relevant features rather than considering only non-redundant features. This was a major reason for choosing Bortura, as it provides a list of all the important features, rather than finding a compact subset of features in which the model achieves higher performance. The algorithm completed 100 independent trials and divided features into 3 parts: confirmed, tentative, and rejected. In our experiment, of the 1088 original features, 381 features were confirmed, and 3 features were tentative. During the experiments, it was found that tentative features contribute to improving the performance of the model, they, along with the confirmed features, were included in model 9.

All models were gradient boosted trees (XGB) as previous work by our group has shown this technique to consistently perform well^3,5^. The main advantage of gradient boosted trees, as opposed to other tree based models, is that trees are created sequentially which reduces the residual error of previous trees and recent work by Yu et al. have shown that XGB XGB attained a balanced performance across accuracy, runtime, and energy efficiency in the medical datasets^24^.

The model was created in Python v 3.8.0. The gradient boosted tree classifiers were implemented using the XGBoost package (version 1.3.3) and the “genism” library was used used in the Word2Vec model. Model hyperparameters were selected using five-fold cross-validation with grid-search on the training dataset, where patients undergoing multiple surgeries appeared only in the training or testing set, but not simultaneously in both. In five-fold cross-validation, the dataset is divided into five partitions; four-fifths of the data is used to train the models and the remaining one-fifth is used as the testing set. This process is repeated so that each partition is used as a testing set only once and a training set four times. Cross-validation provides a better assessment of model performance by averaging metrics across multiple tests. The models best parameters were a maximum depth of 12, and minimum child weight of 5. A copy of the code can be found at https://github.com/maritum/PeriopMortality-Prediction.

### Model performance

Prediction of both mortality and AKI were treated as binary classification problems with highly imbalanced classes. The issue of class imbalance has significant implications for metrics of model performance.

Receiver operating characteristic (ROC) curves are widely used for the estimation of predictive model performance with a binary outcome. ROC curves characterize the trade-off between true positive and false positive rates for the binary classification model by varying the discriminative threshold. However, the false positive rate is affected by the underlying rate of the event and can be deceptive for data with a large skew in the class distribution, thus making ROC curves overly optimistic.

Thus, in addition to ROC curves we considered precision-recall (PR) curves, which summarize the trade-off between true positive rate and positive predictive value by changing the prediction threshold. Positive predictive value (or precision) penalizes a model for a large number of false positives relative to the number of true positives that makes PR curves robust even under imbalanced data. Simultaneously recall, instead of focusing on the number of predicted false positives, penalizes a model for a large number of false negative. The penalties in precision and recall are opposites, making this curve a better metric for model performance with imbalanced data.

Lastly, the F-beta score is a useful metric that calculates the weighted harmonic mean of precision and recall, reaching its optimal value at 1 and its worst value at 0. The beta parameter determines the weight of recall in the combined score (https://scikitlearn.org/stable/modules/generated/sklearn.metrics.fbeta_score.html) and allows data scientists to pick a threshold that optimizes the implementation tradeoffs between precision and recall. Numbers greater than 1 will give increased weight to the recall, while those less than one will give increased weight to the precision.

To examine the performance of the different models we examined model performance with beta values of 1, 2, and3 were chosen. The higher beta values gave more weight to the recall and penalized the number of false negatives. The F-beta score is a threshold metric; thus, scores were calculated for each model based on the selected discriminative threshold that maximizes the F-score. One of the limitations of these metrics is that they assume the distribution of the classes observed in the training dataset will match the distribution in the test set and in real data when the model is used to make predictions.

## Results

Overall the dataset identified 101,070 surgeries across 93,335 admission and 79,662 patients. Patient ages ranged between 18 and 89 years with a mean age of almost 55.79. Overall, the rate of mortality in the dataset was 2.29% and the rate of AKI was 15.8%. ASA Physical status of 3 was the most common physical status and the most common surgical specialty was General Surgery. Detailed information of patient demographics for both the mortality and AKI models is show in Table 1.

**Table 1:** Patient characteristics.

Property	Population
Patients, *n*	79,662
Admissions, *n*	93,335
Surgeries, *n*	101,070
Mortalities, *n *(%)	2312 (2.29)
Kidney failure	15,985 (15.8)
Mean age	55.79 (18–89)
Female patients, *n *(%)	41,062 (51.55)
**ASA physical status, n (%)**	
1	5629 (5.57)
2	34,468 (34.10)
3	47,596 (47.09)
4	11,294 (11.17)
5	713 (0.71)
**Types of surgery, *n *(%)**	
General surgery	20,097 (19.88)
Orthopaedics	15,346 (15.18)
Urology	12,600 (12.47)
Neurosurgery	10,971 (10.85)
Other	41,639 (41.2)

Patient characteristics for the cohort used for training and testing models. Number of patients and percent of the cohort are shown. The selected surgical services represent the top four most frequent surgical services.

### Receiver operating characteristic and precisions-recall performance of the models

**Figure 2:**
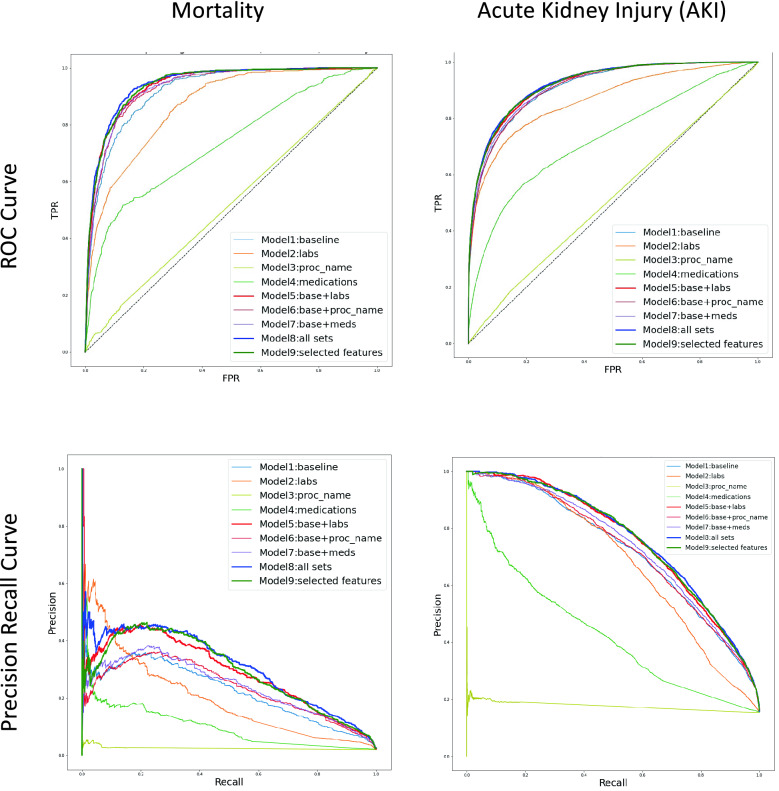
AUC curves.

Table 2 and Fig. 2 demonstrate the performance of the models. Overall, across both outcomes the models that contained combined feature sets (model 8) (Mortality ROC-AUC 94.32 ± 1.01, PR-AUC 36.80 ± 5.10; AKI ROC-AUC 92.45 ± 0.64, PR-AUC 76.22 ± 1.95) was superior to models with only subsets of features and model 9, which contained all features with feature selection (35% of features from model 8), performed nearly as well as model 8.

**Table 2:** Performance metrics for XGBoost model. XGBoost model performance metrics for predicting in-hospital mortality using different sets of features.

	Mortality		AKI	
	ROC-AUC	PR-AUC	ROC-AUC	PR-AUC
Model 1 (baseline)	92.13 ± 0.23	22.93 ± 1.13	91.01 ± 0.54	72.13 ± 1.65
Model 2 (labs)	86.53 ± 0.38	20.03 ± 1.06	86.49 ± 0.48	68.78 ± 0.84
Model 3 (proc_name)	50.04 ± 0.30	3.16 ± 3.11	50.05 ± 0.11	18.12 ± 5.05
Model 4 (medications)	72.26 ± 0.83	9.14 ± 0.58	70.75 ± 0.29	40.06 ± 0.52
Model 5 (baseline ± labs)	92.95 ± 0.25	23.87 ± 1.32	92.10 ± 0.48	75.44 ± 1.87
Model 6 (baseline ± proc_name)	92.89 ± 0.82	27.08 ± 4.12	91.40 ± 0.45	72.79 ± 1.51
Model 7 (baseline ± meds)	93.09 ± 0.22	24.24 ± 1.25	91.37 ± 0.55	73.13 ± 1.62
Model 8 (all sets)	94.32 ± 1.01	36.80 ± 5.10	92.45 ± 0.64	76.22 ± 1.95
Model 9 (all_sets ± feature selection)	93.76 ± 0.95	32.33 ± 5.23	92.32 ± 0.47	75.22 ± 1.71

For the models with the individual feature sets (models 1–4), the baseline feature set, those features selected by clinicians, performed the best with a ROC-AUC of 92.13 ± 0.23 for mortality and 91.01 ± 0.54 for AKI, and a PR-AUC of 22.93 ± 1.13 for mortality and 72.13 ± 1.65 for AKI. In general ROC-AUC tended to be higher for mortality and the PR-AUC was higher for AKI. Figure 1 shows the ROC and PR curves for these models.

In looking at the combined feature sets there were fewer clear themes, but it was notable that even features that had relatively lower AUCs on their own (such as procedure names and medications) seemed to improve the models when added to the baseline features.

### Secondary performance metrics—accuracy, precision and recall and various thresholds

Table 3 shows the overall F1, F2 and F3 scores as well as the accuracy, precision and recall, specificity, and negative predictive value (NPV) at the various thresholds. There were fewer clear trends across the different models with much greater variability in the performance from one model to the next. Of note, Model 3 (procedure name) achieved perfect recall for both outcomes though with very low precision. For the AKI outcome some of the models achieved very high recall (above 90%) with overall accuracies above 75%.

**Table 3:** Performance metrics for XGBoost model using different sets of features.

	Mortality						AKI					
	F1						F1					
	Score	Accurarcy	Recall	Precision	Specificity	NPv	Score	Accuracy	Recall	Precision	Specificity	NPV
Model 1 (baseline)	36.3 ± 2.2	96.6 ± 0.3	45.2 ± 3.6	33.5 ± 2.9	97.7 ± 0.4	98.8 ± 0.1	66.1 ± 1.0	89.5 ± 0.5	67.0 ± 1.4	66.1 ± 1.9	93.5 ± 0.7	94.1 ± 0.2
Model 2 (labs)	48.3 ± 2.9	92.8 ± 0.8	49.1 ± 2.7	50.7 ± 3.5	96.1 ± 0.5	95.7 ± 0.5	63.0 ± 0.8	89.0 ± 0.4	60.7 ± 1.2	66.4 ± 1.8	94.1 ± 0.5	92.9 ± 0.2
Model 3 (proc_name)	4.2 ± 0.4	5.9 ± 5.0	96.3 ± 4.8	3.3 ± 1.9	4.1 ± 5.2		27.1 ± 0.4	15.7 ± 0.3	100.0 ±	15.7 ± 0.3	0.1 ± 0.0	
Model 4 (medications)	22.7 ± 1.7	95.3 ± 0.6	32.1 ± 2.9	21.8 ± 4.0	96.6 ± 0.6	98.6 ± 0.1	46.2 ± 0.9	82.0 ± 0.5	49.8 ± 1.5	43.8 ± 1.5	87.9 ± 0.8	90.5 ± 0.3
Model 5 (baseline + labs)	40.6 ± 2.7	97.1 ± 0.4	50.9 ± 4.8	40.3 ± 4.0	98.0 ± 0.4	99.0 ± 0.1	69.2 ± 0.8	90.6 ± 0.4	67.6 ± 1.1	71.4 ± 1.4	94.8 ± 0.5	94.1 ± 0.2
Model 6 (baseline + proc_name)	37.8 ± 2.9	96.8 ± 0.3	47.7 ± 3.2	34.5 ± 3.9	97.8 ± 0.3	98.9 ± 0.1	66.3 ± 0.8	89.7 ± 0.4	66.9 ± 1.5	66.4 ± 1.4	93.7 ± 0.5	94.1 ± 0.2
Model 7 (baseline + meds)	38.3 ± 2.7	96.8 ± 0.4	46.3 ± 3.8	37.3 ± 3.8	97.9 ± 0.5	98.9 ± 0.1	67.2 ± 0.9	90.1 ± 0.3	66.0 ± 1.3	69.1 ± 1.7	94.5 ± 0.5	93.9 ± 0.2
Model 8 (all sets)	43.8 ± 2.5	97.5 ± 0.2	50.2 ± 3.1	42.3 ± 3.9	98.4 ± 0.2	99.0 ± 0.1	69.4 ± 0.8	90.8 ± 0.2	69.5 ± 1.4	69.9 ± 1.4	94.6 ± 0.4	94.6 ± 0.2
Model 9 (all_sets +feature selection)	41.7 ± 2.9	97.1 ± 0.2	51.2 ± 3.3	37.7 ± 3.7	98.0 ± 0.3	99.0 ± 0.1	68.6 ± 1.0	90.4 ± 0.4	68.6 ± 1.7	69.6 ± 1.9	94.3 ± 0.6	94.4 ± 0.2

	Mortality						AKI					
	F2						F2					
	Score	Accurarcy	Recall	Precision	Specificity	NPv	Score	Accuracy	Recall	Precision	Specificity	NPV
Model 1 (baseline)	49.6 ± 2.3	92.5 ± 0.8	69.8 ± 3.4	19.6 ± 1.8	93.0 ± 0.9	99.3 ± 0.1	75.8 ± 0.7	77.5 ± 1.3	89.0 ± 1.0	40.2 ± 1.5	75.4 ± 1.7	97.5 ± 0.2
Model 2 (labs)	57.0 ± 2.7	85.0 ± 1.7	69.8 ± 2.5	30.0 ± 2.3	85.0 ± 1.9	97.4 ± 0.3	70.8 ± 0.6	78.0 ± 1.5	80.9 ± 0.9	40.7 ± 1.5	77.4 ± 1.9	95.7 ± 0.2
Model 3 (proc_name)	12.9 ± 0.6	4.0 ± 3.6	98.2 ± 3.4	2.3 ± 0.6	2.1 ± 3.7		57.4 ± 0.5	15.7 ± 0.3	100.0 ±	15.7 ± 0.3	0.1 ± 0.0	
Model 4 (medications)	33.0 ± 1.7	91.1 ± 0.9	48.0 ± 2.4	12.7 ± 1.6	92.0 ± 0.9	98.8 ± 0.1	58.8 ± 0.6	31.9 ± 4.5	93.1 ± 2.6	19.0 ± 1.3	20.5 ± 5.8	95.6 ± 0.5
Model 5 (baseline + labs)	55.5 ± 2.6	94.0 ± 0.7	75.4 ± 2.5	23.5 ± 2.4	94.3 ± 0.8	99.5 ± 0.1	78.3 ± 0.6	79.3 ± 1.0	90.6 ± 0.8	42.8 ± 1.1	77.2 ± 1.3	97.9 ± 0.1
Model 6 (baseline + proc_name)	51.2 ± 2.6	93.2 ± 0.8	71.2 ± 2.8	21.9 ± 2.7	93.7 ± 0.9	99.4 ± 0.1	76.1 ± 0.7	78.4 ± 1.2	88.6 ± 0.9	41.3 ± 1.7	76.6 ± 1.5	97.4 ± 0.2
Model 7 (baseline + meds)	52.6 ± 2.6	92.7 ± 0.9	74.2 ± 2.9	21.3 ± 2.4	93.1 ± 0.9	99.4 ± 0.1	76.9 ± 0.7	78.0 ± 1.1	90.0 ± 0.9	40.9 ± 1.3	75.8 ± 1.4	97.7 ± 0.2
Model 8 (all sets)	56.3 ± 2.1	94.4 ± 0.8	74.2 ± 2.6	24.8 ± 2.2	94.8 ± 0.8	99.5 ± 0.1	77.9 ± 0.7	81.8 ± 1.0	88.3 ± 0.9	45.6 ± 1.6	80.6 ± 1.3	97.5 ± 0.2
Model 9 (all_sets +feature selection)	55.7 ± 2.8	94.3 ± 0.6	73.1 ± 3.2	24.8 ± 2.6	94.7 ± 0.7	99.5 ± 0.1	77.5 ± 0.7	80.9 ± 1.4	88.2 ± 1.2	45.0 ± 1.7	79.5 ± 1.8	97.5 ± 0.2

	Mortality						AKI					
	F3						F3					
	Score	Accurarcy	Recall	Precision	Specificity	NPv	Score	Accuracy	Recall	Precision	Specificity	NPV
Model 1 (baseline)	54.1 ± 2.3	90.7 ± 0.8	76.0 ± 2.7	16.6 ± 1.4	91.0 ± 0.9	99.5 ± 0.1	79.3 ± 0.6	73.6 ± 1.6	91.8 ± 1.0	36.6 ± 1.5	70.3 ± 2.0	98.0 ± 0.2
Model 2 (labs)	60.2 ± 2.7	79.5 ± 2.8	74.3 ± 2.8	25.7 ± 2.0	78.1 ± 3.3	97.8 ± 0.3	73.8 ± 0.5	68.5 ± 3.3	86.7 ± 1.5	33.9 ± 2.1	65.1 ± 4.2	96.6 ± 0.2
Model 3 (proc_name)	16.9 ± 0.8	2.1 ± 0.1	100.0	2.0 ± 0.1	0.1 ± 0.0		65.0 ± 0.4	15.7 ± 0.3	100.0	15.7 ± 0.3	0.1 ± 0.0	
Model 4 (medications)	36.2 ± 1.7	90.4 ± 1.0	49.7 ± 2.7	11.8 ± 1.3	91.3 ± 1.0	98.9 ± 0.1	65.8 ± 0.5	23.0 ± 0.9	98.3 ± 0.4	16.6 ± 0.3	9.1 ± 1.1	96.9 ± 0.5
Model 5 (baseline + labs)	59.7 ± 2.5	93.4 ± 0.8	77.3 ± 2.3	22.1 ± 2.3	93.7 ± 0.8	99.5 ± 0.0	81.6 ± 0.5	77.2 ± 1.0	92.3 ± 0.7	40.3 ± 1.1	74.3 ± 1.2	98.2 ± 0.1
Model 6 (baseline + proc_name)	55.4 ± 2.5	92.2 ± 0.9	74.5 ± 2.7	19.4 ± 2.3	92.6 ± 1.0	99.5 ± 0.1	79.6 ± 0.6	72.9 ± 1.4	92.7 ± 0.7	35.8 ± 1.3	69.3 ± 1.8	98.2 ± 0.1
Model 7 (baseline + meds)	57.3 ± 2.5	91.2 ± 0.9	79.4 ± 2.4	18.2 ± 1.9	91.4 ± 1.0	99.5 ± 0.1	80.5 ± 0.6	74.3 ± 1.4	92.9 ± 0.7	37.3 ± 1.4	70.9 ± 1.7	98.2 ± 0.2
Model 8 (all sets)	60.4 ± 2.0	93.4 ± 0.8	78.6 ± 2.5	22.1 ± 2.0	93.7 ± 0.9	99.6 ± 0.1	80.8 ± 0.6	77.6 ± 1.2	91.4 ± 0.7	40.3 ± 1.4	75.1 ± 1.5	98.0 ± 0.1
Model 9 (all_sets +feature selection)	59.7 ± 2.7	93.1 ± 0.8	77.2 ± 2.7	22.7 ± 2.5	93.4 ± 0.9	99.5 ± 0.0	80.6 ± 0.7	75.7 ± 1.6	92.1 ± 0.8	38.9 ± 1.6	72.6 ± 2.0	98.1 ± 0.1

## Discussion

In this manuscript we successfully created feature vectors from a variety of clinical data types (medications, laboratory results, and surgical procedure name) and were able to leverage these additional features to improve the performance of models to predict postoperative AKI and mortality as compared to a baseline feature set chosen by clinician judgment. Of particular note, some features (such as procedure name) which did not necessarily perform well on their own, still enhanced the performance of the models when combined with other features. Additionally, while there were some trends in model performance, the additional benefit of a particular feature often differed between the two outcomes.

We believe that these results lead to some important conclusions. Firstly, these results demonstrate that the inclusion of more information from the electronic health record has the ability to improve model performance. Crucially, for the outcomes in this paper, the improvement in performance was not linear—i.e. more features were not always better, and features that performed poorly in one model performed better when combined with different features in another model. We believe these results point to the need for more research into a variety of modeling techniques. While this manuscript used gradient boosted trees, the performance of other techniques, such as neural networks, might be better or at least different.

Of note, there is far more information in the EHR than what we attempted to include in this manuscript. For example, we focused on a set of commonly used laboratory tests and medications as opposed to all medications and tests. Further, the medications were turned into a binary vector (thus ignoring the dose) and the laboratory results were summarized with basic descriptive statistics. Regarding outcomes, we created models to predict AKI as a binary outcome—however the clinical reality is more complex. In fact the AKIN criteria have 3 stages of acute kidney injury. It is possible that the model we created performs better at predicting more severe injury or that a model performing a multi-class prediction would perform differently. Additional work examining things like the use of medication dose, time series techniques, and/or ratios of different laboratory results may yield even more powerful results. Additionally, use of natural language processing techniques on clinician notes, image processing of radiographic results and other types of data may further improve model performance.

Lastly, the accuracy, precision, recall, specificity and negative predictive value at the various thresholds studies (F1, F2 and F3 score) demonstrate that there is no one perfect model. For example, the use of procedure name had relatively poor AUC results but achieved perfect recall for both outcomes, indicating its potential use in workflows where that is a key performance metrics. Thus, while summary performance metrics, such as AUC, are useful for global purposes the actual workflow and clinical tradeoffs must be evaluated when picking a specific model for clinical care. Critically, this may require the evaluation of different types of features, or modeling techniques in addition to determining the proper threshold.

As we noted in the introduction, the dataset is highly imbalanced (i.e. the rates of the two outcomes are relatively rare). There are a variety of techniques that can be used to optimize one of the above parameters given this imbalance including oversampling and undersampling; we chose not to attempt anyof these techniques to retain focus on the featurization. However, future work should certainly include such attempts as they may improve model performance.

This study does have some limitations. Most significantly, this is a single center trial examining a single type of model (gradient boosted trees) and two clinical outcomes—postoperative mortality and AKI. While the conclusions we draw from these results are likely applicable to other hospitals and may generalize to other modeling techniques or outcomes, this cannot be known for certain. Additionally, as noted, this study only used certain medications and laboratory results. While we believe that we have identified those most common and most likely to influence outcomes, it is possible that the results would have been different with a different medications/tests.

Overall, we believe this study adds to the body of work that demonstrates the need for more research into techniques to improve healthcare model performance. There is likely no “magic bullet” of a perfect model that always performs the best. Rather what is needed is a variety of techniques (featurization, modeling, etc.) that can be called upon for a specific clinical task to find the optimal model for that workflow. What does seem to be clear from this manuscript, is that having ways to access more data is probably better and simply relying on a small set of features thought to be clinically relevant is unlikely to create the best performing model.

## Supplementary Information

Supplementary Table 1.

Supplementary Table 2.

Supplementary Table 3.

## Data Availability

Due to institutional restriction regarding protected health information and patient privacy the data used in this manuscript are not publicly available. Researchers interested in obtaining the data are encouraged to contact the corresponding author who will do his best to facilitate access subject to institutional guidelines.
