# Supplementary material for: Integration of feature vectors from raw laboratory, medication and procedure names improves the precision and recall of models to predict postoperative mortality and acute kidney injury
Source: Sci Rep. 2022 Jun 17;12:10254. doi: 10.1038/s41598-022-13879-7 (PMC9205878; doi:10.1038/s41598-022-13879-7)
Supplement: Supplementary file 1 — Supplementary Table 1. [file 41598_2022_13879_MOESM1_ESM.pdf]

Supplemental Table 1: Distribution of the baseline feature set

**Continuous Variables**	**Count**	**Mean (95% CI)**
ADMSN_SURGERY_NUMBER_W_ANES	101,070	1.18 (1.17-1.18)
ADULT_MDRD_GFR_BLK	82,955	107.98 (107.61-108.36)
ADULT_MDRD_GFR_WHT	82,955	89.24 (88.93-89.55)
AGE	101,070	55.79 (55.7-55.91)
ANES_CASE_MINUTES	100,888	249.56 (248.57-250.55)
ANES_CRNA_MINUTES	23,762	138.30 (136.36-140.23)
ANES_RESIDENT_MINUTES	73,759	246.42 (243.11-249.72)
BMI	99,645	28.63 (27.57-29.69)
COLLOID_ML	15,631	632.90 (624.42-641.38)
CRYSTALLOID_ML	49,131	1106.38 (1097.44-1115.33)
DURATION_MINUTE	101,070	252.52 (250.98-254.07)
HEIGHT_IN	99,359	66.63 (66.61-66.66)
MAX_GLUCOSE	24,185	174.70 (173.88-175.52)
MIN_GLUCOSE	24,185	123.00 (122.5-123.49)
PRIM_SURG_PROV_MINUTES	101,070	151.69 (150.54-152.84)
TOT_RBC	101,070	119.39 (113.92-124.85)
TURNOVER_TIME_ANY_PROV	27,610	58.14 (57.81-58.47)
WEIGHT_KG	100,313	78.40 (78.27-78.53)

Categorical Variables	Count	%
**Anestasia type handoff**		
GENERAL	90,926	90.86%
MAC	3,687	3.90%
MAC WITH LOC	2,863	2.69%
EPIDURAL	1,166	1.22%
SPINAL	932	1.03%
REGIONAL BLOCK	259	0.26%
BIER BLOC	17	2.00%
**Ethnicity**		
NOT HISPANIC OR LATINO	79,596	78.89%
HISPANIC OR LATINO	20,105	19.93%
OTHER	1,200	1.19%
**Location**		
RR OR	67,797	67.19%
SM OR	33,104	32.81%
**Patient class**		
SAME DAY ADMIT	48,813	48.30%
INPATIENT	33,889	33.53%
OVERNIGHT RECOVERY	13,766	13.62%
EMERGENCY	4,599	4.55%

**Binary Variables**	Count	%
ART_LINE	27,844	0.27549
NITRIC_OXIDE	1,514	0.01498
PA_CATHETER	7,238	0.07161
SMOKING_YN	5,925	0.05862
