# Supplementary material for: Integration of feature vectors from raw laboratory, medication and procedure names improves the precision and recall of models to predict postoperative mortality and acute kidney injury
Source: Sci Rep. 2022 Jun 17;12:10254. doi: 10.1038/s41598-022-13879-7 (PMC9205878; doi:10.1038/s41598-022-13879-7)
Supplement: Supplementary file 2 — Supplementary Table 2. [file 41598_2022_13879_MOESM2_ESM.pdf]

Albumin_serum
Alkaline_phosphataseplasma
Alt_plasma
Anion_gap
Ast_plasma
Base_excess
Basophils
Bicarbonate_arterial
Bicarbonate_venous
Bilirubin_direct_plasma
Bilirubin_total_plasma
Calcium_serum
Chloride_serum
Creatinine_serum
Eosinophils
Fibrinogen
Glucose_serum_fasting
Glucose_serum_postprandial
Hdl_plasma
Hematocrit
Hemoglobin_serum
Inr
Ldl_plasma
Lymphocytes
Magnesium_plasma
Monocytes
Neutrophils
Partial_thromboplastin_time
Platelet_count
Potassium_serum
Prealbumin
Prothrombin_time
Sodium_serum
Tsh
Urea_nitrogen_blood
White_blood_cell_count

Supplemental Table 2. List of laboratory tests used in the laboratory features
