# Supplementary material for: Integration of feature vectors from raw laboratory, medication and procedure names improves the precision and recall of models to predict postoperative mortality and acute kidney injury
Source: Sci Rep. 2022 Jun 17;12:10254. doi: 10.1038/s41598-022-13879-7 (PMC9205878; doi:10.1038/s41598-022-13879-7)
Supplement: Supplementary file 3 — Supplementary Table 3. [file 41598_2022_13879_MOESM3_ESM.pdf]

Acetaminophen_inpt
Amiodarone_hm
Amiodarone_inpt
Amitriptyline_inpt
Apixaban_inpt
Aripiprazole_hm
Aspirin_hm
Aspirin_inpt
Atenolol_hm
Atenolol_inpt
Baclofen_inpt
Benzapril_hm
Benzapril_inpt
Bosentan_inpt
Captopril_hm
Carvedilol_inpt
Celecoxib_hm
Citricacid_hm
Codeine_inpt
Cyclobenzaprine_hm
Cyclobenzaprine_inpt
Dabigatran_hm
Dexamethasone_hm
Diazepam_hm
Diazepam_inpt
Digoxin_hm
Diltiazem_hm
Diltiazem_inpt
DuloxetineHydrochloride_hm
DuloxetineHydrochloride_inpt
Enalapril_inpt
Enoxaparin_hm
Enoxaparin_inpt
Epoprostenol_inpt
Eszopiclone_hm
Eszopiclone_inpt
Fentanyl_hm
Fentanyl_inpt
Furosemide_hm
Furosemide_inpt
Gabapentin_inpt
Glipizide_hm
Glucagon_hm
Glucose_hm
HCTZ_hm
Haloperidol_inpt
Heparin_hm
Heparin_inpt
Homatropine_hm
Hydralazine_inpt
Hydrocodone_hm
Hydrocodone_inpt
Hydromorphone_hm
Hydromorphone_inpt
Hydroxyzine_hm
Ibuprofen_hm
Ibuprofen_inpt
Iloprost_hm
Iloprost_inpt
Insulin_hm
Insulin_inpt
Irbesartan_hm
IsosorbideMononitrate_inpt
Macitentan_hm
MagnesiumSulfate_hm
MagnesiumSulfate_inpt
Meperidine_inpt
Metformin_hm
Methadone_hm
Methadone_inpt
Methocarbamol_inpt
Metoclopramide_hm
Metoclopramide_inpt
Metoprolol_hm
Metoprolol_inpt
Midazolam_inpt
Morphine_inpt
Nadolol_inpt
Naproxen_hm
Nebivolol_inpt
Nitroglycerin_inpt
Nortriptyline_hm
Nortriptyline_inpt
Olanzapine_inpt
Ondansetron_hm
Ondansetron_inpt
Oxycodone_inpt
Pioglitazone_hm
Pregabalin_inpt
Propofol_inpt
Propranolol_inpt
QuetiapineFumarate_hm
QuetiapineFumarate_inpt
Riociguat_hm
Rocuronium_inpt
Sacubitril;Valsartan_inpt
ScopolamineHydrobromide_hm
ScopolamineHydrobromide_inpt
SerumAlbumin;Colloid_inpt
SildenafilCitrate_hm
SildenafilCitrate_inpt
SodiumChloride;Crystalloid_inpt
Sodiumcitrate_hm
Sumatriptan_hm
Sumatriptan_inpt
TPN_hm
Tadalafil_hm
Tizanidine_hm
Valsartan_hm
Valsartan_inpt
VenlafaxineHydrochloride_hm
VenlafaxineHydrochloride_inpt
Verapamil_hm
Verapamil_inpt
Warfarin_hm
Zolpidem_hm
Zolpidem_inpt

Supplemental Table 3. Medications taken by the patient before the surgery. Suffix `inpt` define medications that were taken inpatient, suffix `hm` refers to the medications taken at home
